# How a first impression biases cognitive load assessments: Anchoring effects in problem-solving tasks of varying element interactivity

**DOI:** 10.3758/s13421-025-01764-3

**Published:** 2025-07-30

**Authors:** Felix Krieglstein, Manuel Schmitz, Lukas Wesenberg, Markus Wolfgang Hermann Spitzer, Günter Daniel Rey

**Affiliations:** 1 Psychology of Learning with Digital Media, Institute for Media Research, Faculty of Humanities, Chemnitz University of Technology, Chemnitz, Germany; 2 Department of Psychology, Experimental and Applied Neuropsychology, Philipps University of Marburg, Marburg, Germany; 3 Department of Psychology, Martin-Luther University Halle-Wittenberg, Halle, Germany

**Keywords:** Anchoring effect, Cognitive load, Complexity, Heuristics, Problem-solving

## Abstract

The anchoring effect is a cognitive bias in which people rely heavily on an initial piece of information when making judgments or decisions. Once an anchor – typically an objective numerical value – is set, subsequent assessments are adjusted around it, often insufficiently. The extent to which this effect influences cognitive load assessments is unclear. Particularly when students are required to assess cognitive load multiple times during problem-solving, they may resort to heuristics to simplify the cognitively demanding decision-making process. This experimental series aimed to investigate whether anchoring biases cognitive load assessments when students evaluated the cognitive load of several problem-solving tasks. Across three experiments (*N*_1_ = 100, *N*_2_ = 87, *N*_3_ = 80) students assessed the cognitive load of tasks with varying levels of element interactivity (low, moderate, high) multiple times during problem solving. Task sequences were varied to examine whether the first impression of complexity influenced subsequent assessments. The results were mixed: In Experiments 1 and 2, the first impression did not influence the following assessment, but Experiment 3 confirmed the hypothesized anchoring effect. However, this finding cannot be solely attributed to anchoring, as several factors – such as subjective perceptions of complexity, scale effects, task-specific differences, and memory and consistency effects – may have contributed. The findings suggest that anchoring is more likely to occur when there is a substantial contrast between the anchor and subsequent assessments. Furthermore, objective anchors, such as pre-defined numerical values, may exert a stronger influence on decision-making processes than subjective ones, like self-generated assessments.

## Introduction

Cognitive load theory (CLT) is widely recognized as one of the most influential frameworks in educational psychology. The theory is particularly relevant for problem-solving tasks, as it provides insights into how cognitive resources can be optimized to handle complex information effectively. Consequently, several principles and recommendations have been developed, which are predominantly tested through experimental studies measuring cognitive load (Sweller et al., 2011). Typically, this measurement involves asking students to self-assess the cognitive load they experienced during problem solving (Ayres, 2006; Krieglstein et al., 2022). Research highlights the importance of repeated cognitive load assessments throughout problem-solving activities, ideally after each task (Kalyuga et al., 2001; Krieglstein et al., 2024; Paas, 1992; Schmeck et al., 2015; Van Gog et al., 2012).

However, frequent assessments within short time spans may cause cognitive fatigue, particularly when tasks vary in the cognitive load they induce. In such situations, students may resort to heuristics to simplify their assessments. One common heuristic is the reliance on the initial impression as an anchor, which can influence subsequent evaluations – a phenomenon known as the *anchoring effect* (Furnham & Boo, 2011; Tversky & Kahneman, 1974). This tendency is further amplified by the inherently relative nature of subjective cognitive load assessments, which depend on both individual characteristics (e.g., prior knowledge) and task features (e.g., element interactivity; see Brünken et al., 2010).

In this sense, relativity is a general characteristic of cognitive load assessments. The anchoring effect examined here, however, reflects a specific cognitive mechanism within this broader relativity. It describes the systematic influence of the initially experienced task as a reference point for subsequent assessments, regardless of the actual difficulty of the later task. Anchoring is thus not merely a byproduct of general relativity, but a cognitive bias arising from the salience and sequence of prior experiences. To reduce the potential confounding influence of individual differences in prior knowledge, and thereby isolate the anchoring effect more clearly, we employed weekday-problems (Schmeck et al., 2015; Van Gog et al., 2012). It can reasonably be assumed that all participants had comparable knowledge regarding the sequence of weekdays. This design choice was intended to keep prior knowledge as constant as possible across participants.

To investigate the proposed anchoring effect, three experiments were conducted to examine whether students’ initial cognitive load impressions influenced their subsequent judgments during problem solving. The tasks were systematically manipulated based on element interactivity to induce varying levels of cognitive load. While most empirical studies on anchoring focus on objective anchors (e.g., externally provided numerical values), the present research addresses whether subjective impressions can elicit a similar bias in the context of cognitive load assessment. This approach is more closely aligned with experimental practice, where students typically assess their cognitive load intuitively and without externally provided reference points.

One of the main assumptions of CLT is that problem solving – defined as the cognitive process of applying knowledge, strategies, and reasoning to reach a desired solution – is impaired when the cognitive load imposed by the task exceeds the available cognitive resources (Sweller, 1988). Consequently, CLT aims to provide recommendations and principles to help students use their cognitive resources effectively for processes relevant to problem solving (Renkl et al., 2004). When trying to understand why students were (or were not) able to successfully complete a problem-solving task, the concept of intrinsic cognitive load (ICL) offers a valuable explanatory approach. ICL refers to the inherent complexity of the task, which is determined by the concept of element interactivity (Chen et al., 2023). Element interactivity is defined as the number of interacting elements within the task that must be processed simultaneously in working memory (Sweller, 2010). Problem-solving tasks can therefore be classified along a continuum from low to high element interactivity.

This becomes particularly clear when examining the following two linear equations, which can be defined as problem-solving tasks (Star & Rittle-Johnson, 2008). The linear Eq. 2x = 40 involves lower element interactivity compared to the Eq. 2x + 12 = 30–4x. This is primarily because the first equation requires processing fewer elements (three elements when 2 × is considered as one), whereas the second equation involves more elements (seven elements in total). Thus, solving the second task imposes a higher working memory load, as more elements must be processed simultaneously. Adding additional elements further increases the element interactivity of the task and, consequently, complexity^1^ and ICL. The same applies to text-based tasks, where inserting additional words that require simultaneous processing increases element interactivity (e.g., weekday problems; Sweller, 1993).

As previously noted, research has emphasized the importance of measuring cognitive load after each problem-solving task, particularly when multiple tasks are involved. This approach allows for more precise measurements (Kalyuga et al., 2001; Krieglstein et al., 2024; Paas, 1992; Schmeck et al., 2015; Van Gog et al., 2012). However, repeated cognitive load assessments can be cognitively exhausting for students, as they must evaluate and report their perceived cognitive load in addition to solving the actual problem. Assessing cognitive load can be defined as a metacognitive task since students must continuously monitor their cognitive processes in relation to task demands (Seufert, 2018). Therefore, students must not only solve the problem, but also assess their cognitive load during the process. This dual requirement is a complex task that becomes particularly challenging when the problem-solving task is difficult to solve (Bratfisch et al., 1972) or when students lack the metacognitive skills to assess cognitive load retrospectively.

In this context, students may seek ways to simplify the complex process of assessing cognitive load during problem solving. One possible approach is the use of heuristics, defined as mental rules of thumb or simplifications (Gigerenzer & Gaissmaier, 2011). Heuristics enable individuals to make complex decisions quickly and with minimal cognitive effort. However, decisions based on heuristics may overlook relevant information and can be biased (Thorngate, 1980). People tend to rely on heuristics when seeking cognitive efficiency (Shah & Oppenheimer, 2008), when lacking sufficient information to make an informed decision, leading to uncertainty (Dosi & Egidi, 1991), or when facing time constraints (Bobadilla-Suarez & Love, 2018).^2^

A well-researched heuristic is *anchoring*, a cognitive bias in which people heavily rely on an initial piece of information (i.e., the “anchor”) when making subsequent judgments or decisions (Tversky & Kahneman, 1974). This initial piece of information is established as a reference point, and people try to make adjustments from this anchor in subsequent assessments (Epley & Gilovich, 2006). Since adjustments are cognitively complex or influenced by uncertainty and time pressure, the assessments often remain close to the anchor. Epley and Gilovich (2006) emphasize that these adjustments often occur automatically and unconsciously, leading to insufficient corrections. The two cognitive processes (anchoring and adjustment) result in the designation *anchoring-and-adjustment heuristic*. For example, in an experiment by Tversky and Kahneman (1974), participants were asked to estimate various quantities, such as the percentage of African countries in the United Nations. Before estimating the percentage, participants were presented with a seemingly relevant but objectively meaningless number generated by a wheel of fortune, which stopped at either 10 or 65. Participants whose wheel stopped at 10 estimated lower values (median of 25%) than participants whose wheel stopped at 65 (median of 45%). This demonstrates how even arbitrary anchors can significantly influence numerical judgments.

Research on anchoring effects in human decision-making processes is largely based on studies in which individuals are presented with uninformative (or even implausibly extreme) anchor values before answering subsequent questions in various domains (Furnham & Boo, 2011; Mussweiler, 2001). In most cases, the anchor is a concrete numerical value, as in the study by Tversky and Kahneman (1974). For example, McElroy and Dowd (2007) asked participants to estimate whether the length of the Mississippi River is more or less than 200 or 20,000 miles. In a study by Mussweiler and Englich (2005), participants were asked to estimate the annual mean temperature in Germany. Before making this decision, they were presented with an anchor that was either low (5 °C) or high (20 °C).

Such objective manipulations allow potential anchoring effects on subsequent judgments to be investigated experimentally in a controlled manner. For example, in the study by Tversky and Kahneman (1974), the objective numerical value presented before decision making (i.e., estimating the percentage of African countries in the United Nations) was assumed to affect subsequent judgments. However, subjective estimates can also serve as anchors for subsequent judgments. This was demonstrated in a study by Goller et al. (2018) on the perception of facial attractiveness. Given that first impressions shape subsequent behavior, participants were presented with pictures of the same person in ascending (low-to-high) or descending (high-to-low) order of attractiveness. Consistent with the anchoring effect, the results revealed that subsequent attractiveness ratings were higher for the descending condition than for the ascending condition. Overall, this suggests that assessments of attractiveness, which are highly subjective, are also influenced by first impressions and the sequence in which different levels are presented.

Similarly, this work examined whether self-assessments of cognitive load, made by students when they are asked to assess it multiple times across several problem-solving tasks, are biased towards the first impression of cognitive load. There is a lack of empirical evidence on whether the anchoring heuristic operates during cognitive load assessments. Given that cognitive load is often measured during learning or problem solving (Ayres, 2006; Krieglstein et al., 2022; Leppink & Van Merriënboer, 2015), research on anchoring in these settings might be helpful for deriving initial assumptions regarding anchoring in cognitive processes.

In the field of metacognition and learning, empirical evidence suggests that students’ judgments of learning (JOL) – their self-assessed likelihood of recalling studied material in a test – can be influenced by anchor values (Ikeda, 2023; Ikeda et al., 2024; Yang et al., 2018). For instance, Ikeda (2023) asked participants to predict their performance in an upcoming memory test. Specifically, participants were instructed to assess whether their performance would be higher or lower than a given anchor (80% in the high-anchor condition or 20% in the low-anchor condition). Following this pre-study prediction, they learned 20 word pairs and made a post-study prediction. The results indicated that both pre- and post-study predictions were significantly higher in the high-anchor condition, demonstrating an uninformative anchoring effect, as the anchor value should not have influenced these predictions.

Another facet of metacognition is the allocation of study time, which refers to the process by which students distribute their available time and mental resources to achieve optimal learning. Previous research has shown that external factors (e.g., advice from teachers) can influence study-time allocation. In this context, Li et al. (2024) investigated whether providing students with information about the average time other students spent studying each word pair affected study time and recall performance. Building on the anchoring effect, the instruction specified either a shorter time period (5 s in the low-anchor condition) or a longer time period (15 s in the high-anchor condition). The results supported the hypothesized anchoring effect, as indicated by longer study times in the high-anchor condition. Additionally, students in the high-anchor condition demonstrated higher recall performance. Taken together, these findings suggest that anchoring effects influence students’ metacognitive judgments and their organization of study time. However, it is important to note that these studies rely exclusively on objective, pre-defined numerical values as anchors.

It remains unclear whether the process of assessing cognitive load multiple times during problem solving is influenced by anchoring effects. Given the complexity of this assessment, it is plausible that students resort to heuristics, as the process is resource intensive (Lieder et al., 2018). Research on mental effort avoidance and the cost of cognitive effort supports this notion, showing that people generally prefer low-effort tasks (Embrey et al., 2023; Inzlicht et al., 2018). Based on this assumption, students may be particularly susceptible to anchoring effects, as heuristics help minimize cognitive resource demands. Additionally, the assessment of cognitive load inherently involves a degree of uncertainty (Hadfi & Ito, 2013).

Similar to the experiment conducted by Goller et al. (2018), this experimental series assumes that anchors can also be based on subjective impressions, such as the perception of complexity, which subsequently influences cognitive load assessments. Therefore, the aim of this series is to better understand the underlying mechanisms involved when students evaluate the complexity (as a facet of cognitive load) of problem-solving tasks with varying levels of element interactivity within a task environment.

The main assumption is that an anchoring effect occurs, meaning that the initial impression of complexity biases subsequent assessments during problem solving. To investigate this effect, students were asked to rate ICL (i.e., as a facet of cognitive load) multiple times without being explicitly provided with an anchor value. In each of the three experiments in this series, the sequence of three problem-solving tasks was manipulated: students initially encountered different tasks (i.e., with varying levels of element interactivity), while the same task (i.e., with identical element interactivity) was consistently presented in the middle. This middle task served as the basis for testing the anchoring hypothesis. Experiment 1 tested the following hypothesis:

*Students in the condition with increasing complexity assess the ICL of the subsequent task unit*^3^* as lower than students in the condition with decreasing complexity.*

## Experiment 1

In the first experiment, students completed three problem-solving tasks that differed in element interactivity. The tasks were presented in an increasing or decreasing sequence of complexity. After completing each problem-solving task, students were asked to report its complexity using an ICL scale. The aim was to determine whether students would base their directly subsequent assessment on the anchor, which is the first impression of complexity.

### Participants and design

Participants in Experiment 1 were recruited through Prolific (https://www.prolific.com/). Three screening criteria were defined to ensure a homogeneous sample. Participants had to live in Germany, be fluent in German, and be enrolled in an undergraduate or graduate program. A total of 100 students (*M*_age_ = 25.09 years, *SD*_age_ = 4.57 years) participated in Experiment 1, 59% of whom were enrolled in an undergraduate program. Of the participants, 39% identified themselves as female, 57% as male, and 4% did not specify their gender. Participants were studying between their first and ninth semester (*M* = 2.72, *SD* = 1.77). They were randomly assigned to one of two conditions (complexity sequence: increasing complexity vs. decreasing complexity) in a between-subjects design. Accordingly, participants solved the three problem-solving tasks of either increasing (sequence of the task units: 1–2 – 3; *n* = 54) or decreasing complexity (sequence of the task units: 3–2 – 1; *n* = 46). In both conditions, the moderate complex task unit was presented in the middle.

### Instructional material

The instructional material consisted of three problem-solving tasks in which students must perform a series of actions to get from state A (initial state) to state B (goal state) without being instructed as to which individual steps are necessary in the process (Newell & Simon, 1972). In concrete terms, students were asked to solve three weekday problems (Schmeck et al., 2015; Sweller, 1993; Van Gog et al., 2012) that differed in their element interactivity (i.e., complexity; see Appendix A). These problem-solving tasks in this sequence have been used in previous studies to manipulate element interactivity (e.g., Schmeck et al., 2015). All participants were presented with the same problem-solving tasks, but in a different sequence depending on the experimental condition. That is, the increasing complexity group was presented the following sequence: task unit 1, task unit 2, and task unit 3. The decreasing complexity group performed the reversed complexity sequence: task unit 3, task unit 2, and task unit 1.

### Measure of complexity

Complexity (resulting from element interactivity) was measured three times during problem solving using the cognitive load questionnaire developed by Krieglstein et al. (2023). Specifically, four items from the ICL scale were used (see Appendix B). The fifth item of the scale was omitted. This decision was based on the fact that this item addresses the interplay between the two core components of intrinsic cognitive load, task complexity and learners’ prior knowledge, and therefore did not fit well with the nature of our tasks. The weekday problems were intentionally designed to vary in element interactivity while relying on universally known content (i.e., the days of the week), thus holding prior knowledge constant. Including this item would likely have introduced noise rather than meaningful variance into the ICL measurement.

All items were presented on a 9-point Likert scale (1 = *not at all applicable*, 9 = *fully applicable*). The selection of this scale width was informed by the development and validation of the questionnaire using a 9-point Likert scale. Furthermore, a meta-analysis conducted by Krieglstein et al. (2022) demonstrated that 9-point scales yield satisfactory reliability. After each problem-solving task, students were asked to assess the respective task unit with regard to ICL. The internal consistency of the ICL assessments across the three tasks was satisfactory, ω = [0.75, 0.94].

### Task performance

Whether the participants solved the problem-solving tasks correctly was also checked. Therefore, either zero (incorrect solution) or one point (correct solution) was given.

### Procedure

The experiment was conducted online in Limesurvey (https://www.limesurvey.org/de). Participants were invited by Prolific to participate. They could start the experiment on their own. By clicking on a link, participants were randomly assigned to one of the two experimental conditions. Within the task environment, participants worked on the three problem-solving tasks (i.e., task units). After each task unit, participants completed the ICL scale. Finally, participants provided some demographic information. In total, the experiment took about 15 min.

### Data analysis

Data were analyzed with JASP version 0.19.3 (JASP Team, 2025). Descriptive statistics for the ICL assessments are presented in Table 1. First, task performance was analyzed descriptively by examining the percentage of participants who correctly solved each task. This served to explore whether the intended differences in element interactivity were reflected in participants’ task performance. Moreover, correlations between task performance and ICL were calculated separately for each task unit and for both complexity sequences. This was done to explore whether lower (or higher) perceived task complexity was associated with higher (or lower) task performance.

**Table 1:** Means and standard deviations for the complexity of the task units (Experiment 1)

	Increasing complexity (*N* = 54)			Decreasing complexity (*N* = 46)	
	*M*	*SD*		*M*	*SD*
Task unit 1	1.28	0.74	Task unit 3	4.65	1.84
Task unit 2	3.03	1.84	Task unit 2	3.17	1.84
Task unit 3	5.01	2.11	Task unit 1	1.58	1.10

*Note*. The ICL items were rated on a 9-Point Likert scale

Second, whether the three problem-solving tasks differed in perceived task complexity was checked. Since the experimental anchor was subjective, it was essential to ensure significant differences in perceived complexity. To this end, a mixed-design repeated-measures analysis of variance (ANOVA) was conducted, with complexity sequence as the between-subjects factor and the three ICL assessments (resulting from the three problem-solving tasks) as the within-subjects factor. This type of analysis assumes equal variances of the differences between all levels (i.e., sphericity), which was tested using Mauchly’s test. In cases where this assumption was violated, either the Greenhouse–Geisser correction (for ε ≤ 0.75) or the Huynh–Feldt correction (for ε > 0.75) was applied (Lane, 2016). In the case of a significant ANOVA, Bonferroni post hoc tests were conducted to compare the three ICL assessments separately for each condition.

Third, we examined whether the two conditions differed in terms of the ICL for the second task unit, thereby testing the hypothesized anchoring effect. A one-tailed two-sample *t*-test was conducted to assess this difference. Prior to the analysis, the assumption of variance homogeneity (using Levene’s test) was checked. In cases where this assumption was violated, Welch’s *t*-test for unequal variances was applied. Due to the central limit theorem, the *t*-test is generally robust to moderate violations of normality when sample sizes are sufficiently large (*n* > 30 per group; Kwak & Kim, 2017). Given the complementary value of frequentist and Bayesian approaches (Fornacon-Wood et al., 2022), the Bayes factor (BF10) was computed to evaluate the strength of evidence for the alternative hypothesis relative to the null hypothesis (Dienes, 2014). The interpretation of Bayes factors followed the guidelines proposed by van Doorn et al. (2021).

#### Results

##### Task performance

Descriptive statistics, presented in Table 2, indicated that task performance varied depending on the task unit. While the lower-element interactivity task was solved correctly by all participants, the percentage of correct answers decreased as element interactivity increased in both complexity sequences. Most correlations between task performance and ICL did not reach statistical significance.

**Table 2:** Percentage of correctly solved tasks by all participants and correlations with intrinsic cognitive load (ICL) (Experiment 1)

	Increasing complexity			Decreasing complexity	
	Percentage	Correlation with ICL		Percentage	Correlation with ICL
Task unit 1	100%	-	Task unit 3	54.3%	−0.38**
Task unit 2	77.8%	0.08	Task unit 2	65.2%	0.01
Task unit 3	68.5%	−0.14	Task unit 1	100%	-

*Note*. The point-biserial correlation coefficient is given, as the task performance is dichotomous. For task unit 1 in both conditions, no correlation could be calculated, as the task performance was constant, i.e., everyone solved the task correctly. *p < 0.05, **p < 0.01, ***p < 0.001

##### Differences in task complexity

Since the assumption of sphericity was violated (Mauchly’s test: *p* = 0.025, ε = 0.93), the Huynh–Feldt correction was applied to adjust the degrees of freedom. The analysis revealed a significant interaction between complexity sequence and the three ICL assessments, *F*(1.90, 186.01) = 162.69, *p* < 0.001, ηp^2^ = 0.62. This result indicated that the pattern of ICL assessments across the three tasks depends on the complexity sequence. Post hoc tests revealed significant differences between all three ICL assessments within each condition (all *p*_Bonf_ < 0.001).

##### Anchoring hypothesis

Since the assumption of variance equality was not violated (Levene’s test:* p* = 0.604), no correction of the degrees of freedom was necessary. The analysis revealed no significant difference between the increasing and decreasing complexity conditions regarding the ICL assessment for the middle task unit (task unit 2), *t*(98) = 0.38, *p* = 0.351, *d* = 0.08, 95% CI [−0.25, 0.41]. The Bayes factor (BF10 = 0.29) indicated moderate evidence in favor of the null hypothesis, suggesting that the data are approximately 3.45 times more likely under the null than under the alternative hypothesis. Thus, the data provide some evidence against the anchoring hypothesis, rather than in favor of it.

#### Discussion

The results of Experiment 1 indicated that the initial impression of complexity (i.e., experienced in the first task unit) does not directly influence the subsequent assessment, thereby failing to support the hypothesized anchoring effect. This was evidenced by the fact that the two conditions of increasing and decreasing complexity did not differ significantly in terms of ICL assessments for task unit 2, which was placed in the middle position in both conditions. Regardless of whether participants first encountered a low-complexity task (i.e., task unit 1) or a high-complexity task (i.e., task unit 3), they assessed the subsequent task unit similarly in terms of complexity, showing no influence from their first impression. It appears that students are not influenced by their initial impression of complexity when the tasks are presented in a linearly increasing or decreasing complexity sequence.

## Experiment 2

After Experiment 1 showed that the first impression of complexity does not influence the immediately following assessment, the sequence of complexity was changed in Experiment 2. This was done to explore all possible combinatorial arrangements of the three tasks. It also allows for greater contrasts between the anchor and subsequent task in terms of element interactivity after presenting the tasks in increasing and decreasing sequence of complexity in Experiment 1. For this purpose, in Experiment 2, the high complex task unit (i.e., task unit 3) was placed in the middle. Correspondingly, the position of task units 1 and 2 was varied in the two conditions so that either task unit 1 or task unit 2 was presented first. Given that Experiment 1 revealed that the first impression of complexity has no influence on the immediately following assessment, this was tested again in Experiment 2. This is intended to rule out the possibility that the result from the first experiment is due to a beta error, and at the same time to verify that the presumed anchoring effect on the subsequent judgments does not occur even with a different complexity sequence. Therefore, the following anchoring hypothesis was formulated:

*Students in the condition with the low complex task unit at the beginning assess the ICL of the task unit in the middle as lower than students in the condition with the low complex task unit at the end.*

### Participants and design

As in Experiment 1, participants were recruited through Prolific. Therefore, the same screening criteria were defined to ensure a homogeneous sample (participants had to live in Germany, be fluent in German, and be enrolled in an undergraduate or graduate program). In addition, participants who participated in the first experiment were excluded. Eighty-seven students (*M*_age_ = 24.92 years, *SD*_age_ = 4.71 years) participated in Study 2, 60% of whom were enrolled in an undergraduate program. Of the participants, 30% identified themselves as female, 69% as male, and 1% did not specify their gender. Participants were studying between their first and 15th semester (*M* = 4.56, *SD* = 3.26). They were randomly assigned to one of two conditions (complexity sequence: low complex task unit at the beginning vs. low complex task unit at the end) in a between-subjects design. Accordingly, participants solved the three problem-solving tasks either with the low complex task unit at the beginning (sequence of the task units: 1–3 – 2; *n* = 46) or with the low complex task unit at the end (sequence of the task units: 2–3 – 1; *n* = 41). In both conditions, the high complex task unit was presented in the middle.

### Instructional material

The instructional materials were identical to Experiment 1, the only difference being that the three task units were presented in two different sequences.

### Measure of complexity

The same four items with identical instructions as in Experiment 1 were used. Again, ICL was measured three times during problem solving. The internal consistency across all ICL assessments was satisfactory ω = [0.81, 0.93].

### Task performance

Again, it was checked whether the participants solved the problem-solving tasks correctly.

### Procedure

The procedure was identical to Experiment 1.

### Data analysis

The same analysis strategy was employed as in Experiment 1. Descriptive statistics for the ICL assessments are presented in Table 3.

**Table 3:** Means and standard deviations for the complexity of the task units (Experiment 2)

	Low complex task unit at the beginning (*N* = 46)			Low complex task unit at the end (*N* = 41)	
	*M*	*SD*		*M*	*SD*
Task unit 1	1.59	1.14	Task unit 2	2.84	1.40
Task unit 3	5.25	1.81	Task unit 3	5.36	2.01
Task unit 2	3.41	1.63	Task unit 1	1.27	0.53

*Note*. The ICL items were rated on a 9-Point Likert scale

#### Results

##### Task performance

Descriptive statistics, presented in Table 4, indicate that task performance varied as a function of task unit, but only in the condition where the low-complexity task appeared at the end. In contrast, in the condition where the low-complexity task was presented first, task units 2 and 3, which differed in element interactivity, were solved correctly at similar rates. Task performance and ICL were negatively correlated in one task unit in each of the two conditions.

**Table 4:** Percentage of correctly solved tasks by all participants and correlations with intrinsic cognitive load (ICL) (Experiment 2)

	Low complex task unit at the beginning			Low complex task unit at the end	
	Percentage	Correlation with ICL		Percentage	Correlation with ICL
Task unit 1	97.8%	−0.45**	Task unit 2	68.3%	0.20
Task unit 3	63.0%	0.14	Task unit 3	58.5%	−0.37*
Task unit 2	63.0%	0.15	Task unit 1	100%	-

*Note*. The point-biserial correlation coefficient is given, as the task performance is dichotomous. For task unit 1 in the condition with the low complex task unit at the end, no correlation could be calculated, as the task performance was constant, i.e. everyone solved the task correctly. *p < 0.05, **p < 0.01, ***p < 0.001

##### Differences in task complexity

Since the assumption of sphericity was violated (Mauchly’s test: *p* = 0.014, ε = 0.91), the degrees of freedom were corrected. The mixed-design repeated-measures ANOVA revealed a significant interaction between complexity sequence and the three ICL assessments, *F*(1.86, 158.28) = 46.14, *p* < 0.001, ηp^2^ = 0.35. This result indicated that the pattern of ICL assessments across the three tasks depends on the complexity sequence. Post hoc tests revealed significant differences between all three ICL assessments within each condition (all *p*_Bonf_ < 0.001).

##### Anchoring hypothesis

No correction of the degrees of freedom was necessary (Levene’s test: *p* = 0.273). The analysis revealed no significant difference between the condition with the low complex task unit at the beginning and the condition with the low complex task unit at the end regarding the ICL assessment for the middle task unit (i.e., task unit 3), *t*(85) = 0.27, *p* = 0.395, *d* = 0.06, 95% CI [−0.30, 0.42]. The Bayes factor (BF10 = 0.28) indicated moderate evidence in favor of the null hypothesis, suggesting that the data are approximately 3.57 times more likely under the null than under the alternative hypothesis. Thus, the data provide some evidence against the anchoring hypothesis, rather than in favor of it.

#### Discussion

In Experiment 2, the sequence of task complexity was manipulated so that students either received the low-complexity or the moderate-complexity task unit first, while the high-complexity task unit was placed in the middle in both conditions. The finding from Experiment 1, which indicated that subsequent complexity assessments were not influenced by initial impressions, was replicated. This was demonstrated by the absence of a significant difference between the two conditions for task unit 3. Regardless of whether students first encountered a less complex task (i.e., task unit 1) or a moderately complex task (i.e., task unit 2), they assessed the subsequent task unit similarly, showing no influence from their initial impression.

## Experiment 3

Although Experiments 1 and 2 did not find evidence for an anchoring effect at the inferential statistical level, the descriptive values in both experiments suggested that the task unit presented in the middle – immediately following the anchor – was assessed as more complex when the first task unit (i.e., the anchor) involved higher element interactivity. Based on this observation and in line with assumptions about the anchoring effect, it is hypothesized that the middle task unit will be rated as more complex if the first task involves high element interactivity. To test this hypothesis, the positions of task units 2 and 3 were varied across conditions so that either task unit 2 or task unit 3 was presented first, with task unit 1 consistently placed in the middle. Therefore, the following anchoring hypothesis was formulated:

*Students in the condition with the high complex task unit at the beginning assess the ICL of the task unit in the middle as higher than students in the condition with the high complex task unit at the end.*

### Participants and design

As in Experiments 1 and 2, participants were recruited through Prolific. Therefore, the same screening criteria were defined to ensure a homogeneous sample (participants had to live in Germany, be fluent in German, and be enrolled in an undergraduate or graduate program). Moreover, participants who participated in the first and second experiment were excluded. Eighty students (*M*_age_ = 23.65 years, *SD*_age_ = 3.69 years) participated in Experiment 3, 62.5% of whom were enrolled in an undergraduate program. Of the participants, 42.5% identified themselves as female, 55% as male, and 2.5% did not specify their gender. Participants were studying between their first and 18th semester (*M* = 4.28, *SD* = 3.27). They were randomly assigned to one of two conditions (complexity sequence: high complex task unit at the beginning vs. high complex task unit at the end) in a between-subjects design. Accordingly, participants solved the three problem-solving tasks either with the high complex task unit at the beginning (sequence of the task units: 3–1 – 2; *n* = 40) or with the high complex task unit at the end (sequence of the task units: 2–1 – 3; *n* = 40). In both conditions, the low complex task unit was presented in the middle.

### Instructional material

The instructional materials were identical to Experiments 1 and 2, the only difference being that the three task units were presented in two different sequences.

### Measure of complexity

The same four items with identical instructions to those in the first and second experiment were used. Again, ICL was measured three times during problem solving. The internal consistency across all ICL assessments was satisfactory ω = [0.70, 0.91].

### Task performance

Again, whether the participants solved the problem-solving tasks correctly was checked.

### Procedure

The procedure was identical to Experiments 1 and 2.

### Data analysis

A similar analysis strategy was used as in Experiments 1 and 2. Descriptive statistics for the ICL assessments are presented in Table 5.

**Table 5:** Means and standard deviations for the complexity of the task units (Experiment 3)

	High complex task unit at the beginning (N = 40)			High complex task unit at the end (N = 40)	
	*M*	*SD*		*M*	*SD*
Task unit 3	5.06	1.74	Task unit 2	2.53	1.11
Task unit 1	1.73	0.87	Task unit 1	1.30	0.56
Task unit 2	3.53	2.11	Task unit 3	5.68	1.86

*Note*. The ICL items were rated on a 9-Point Likert scale

#### Results

##### Task performance

Descriptive statistics, presented in Table 6, indicate that task performance varied as a function of task unit. The task with low element interactivity was solved correctly by all participants, whereas the task with high element interactivity yielded the lowest rate of correct responses. This was shown in both conditions. The task involving moderate element interactivity fell in between. However, none of the correlations between task performance and ICL reached statistical significance.

**Table 6:** Percentage of correctly solved tasks by all participants and correlations with ICL (Experiment 3)

	High complex task unit at the beginning			High complex task unit at the end	
	Percentage	Correlation with ICL		Percentage	Correlation with ICL
Task unit 3	40.0%	−0.05	Task unit 2	70.0%	−0.12
Task unit 1	100%	-	Task unit 1	100%	-
Task unit 2	62.5%	0.02	Task unit 3	52.5%	−0.07

*Note*. The point-biserial correlation coefficient is given, as the task performance is dichotomous. For task unit 1 in both conditions, no correlation could be calculated, as the task performance was constant, i.e. everyone solved the task correctly. *p < 0.05, **p < 0.01, ***p < 0.001

##### Differences in task complexity

The assumption of sphericity was not violated (Mauchly’s test: *p* = 0.117). The mixed-design repeated-measures ANOVA revealed a significant interaction between complexity sequence and the three ICL assessments, *F*(2, 156) = 75.82, *p* < 0.001, ηp^2^ = 0.49. This result indicated that the pattern of ICL assessments across the three tasks depends on the complexity sequence. Post hoc tests revealed significant differences between all three ICL assessments within each condition (all *p*_Bonf_ < 0.001).

##### Anchoring hypothesis

Since the assumption of equal variance was violated (Levene’s test:* p* < 0.001), a Welch *t*-test was conducted. The condition with the high complex task unit at the beginning assessed the ICL of task unit 1 significantly higher than the condition with the high complex task unit at the end, *t*(66.43) = 2.64, *p* = 0.005, *d* = 0.59, 95% CI [0.21, 0.97]. The Bayes factor (BF10 = 8.84) indicated moderate evidence for the alternative hypothesis, suggesting that the data are approximately 8.84 times more likely under the alternative than under the null hypothesis. Thus, the data provide moderate evidence in favor of the anchoring hypothesis.

#### Discussion

Experiment 3 provides further evidence for the rather inconclusive findings regarding anchoring effects in cognitive load assessment. In this experiment, the sequence of task complexity was manipulated so that the task unit with low element interactivity was consistently placed in the middle, while students were presented with either task unit 2 or task unit 3 first. In contrast to Experiments 1 and 2, the first impression of complexity had a significant influence on the assessment of the subsequent task unit. This confirms the proposed anchoring effect, such that students who were first presented with the task unit with high element interactivity (i.e., task unit 3) assessed the ICL of the immediately following task unit significantly higher than students who were first presented with the moderate complex task unit (i.e., task unit 2). Thus, the task unit with high element interactivity acts as an anchor for assessing the complexity of the subsequent task unit.

## General discussion

### Theoretical contributions

The aim of this experimental series was to gain deeper insights into the cognitive mechanisms involved in assessing cognitive load multiple times during problem solving. Previous studies have shown that metacognitive judgments and study time allocations are biased toward an anchor value (Ikeda, 2023; Ikeda et al., 2024; Li et al., 2024; Yang et al., 2018). However, these studies focused on objective anchor values. In contrast, this experimental series investigated whether students use their initial impression of cognitive load as an anchor for subsequent assessments. To test this assumption, students completed three problem-solving tasks that differed in element interactivity. The sequence of tasks was manipulated depending on the experimental condition. Experiments 1 and 2 found no evidence of an anchoring effect on immediately subsequent assessments. Both experiments showed that the initial impression of complexity (i.e., the assumed anchor) resulting from the element interactivity of the initial task had no influence on the complexity assessment of the subsequent task presented in the middle of the sequence.

In contrast, the hypothesized anchoring effect was found in Experiment 3. The assessment immediately following was influenced by the initial impression, as task unit 1 was assessed significantly more complex when students had previously encountered the task unit with high element interactivity (and thus high complexity). This finding suggests two possible interpretations. On the one hand, the anchoring effect appears to occur when students are initially presented with a task perceived as being more complex. On the other hand, the observed effect may not be driven solely by the relative task sequence but rather by the magnitude of the differences between the first two tasks. This could explain the absence of an effect in Experiment 1, where the complexity differences were relatively small. However, this interpretation is challenged by Experiment 2, where no anchoring effect was found, even though the complexity difference was similar, except that the task units were presented in reverse sequence (i.e., task unit 1 was presented first).

Moreover, the results of Experiment 3 may not necessarily reflect an anchoring effect but rather a “bottom effect” in the use of the scale. To respond coherently, students must assign lower ratings to easier tasks and higher ratings to more complex ones. Since task unit 2 (moderate complexity) is already perceived as relatively easy, students may feel compelled to assign very low values to the subsequent task unit 1 (low complexity). Conversely, if task unit 3 (high complexity) is presented first, students have more flexibility to provide reasonable assessments afterward, as they may want to avoid assigning the lowest possible rating in case an even easier task follows. This interpretation is supported by the fact that the scale is broad, with nine levels, offering ample room for adjustment.

Assuming that students perceive complexity as negative, the results of Experiment 3 could be interpreted to suggest that the negative experience of a high-complexity task had a stronger impact on subsequent assessments than a moderate-complexity task. This interpretation aligns with the findings of Baumeister et al. (2001), who proposed that negative events are processed more thoroughly than positive ones. In contrast, when the anchor was a low-complexity task (in Experiments 1 and 2), no anchoring effects were observed, likely because the impression of low complexity was not sufficiently impactful to influence subsequent assessments.

In general, all these possible interpretations suggest that the observed effect cannot be clearly attributed to a classic anchoring effect. Instead, several factors could be involved, including subjective perceptions of complexity, scale effects, task-specific differences, and memory and consistency effects. The results seem to indicate that objective anchors exert a much stronger influence on decision-making processes than subjective ones, as the latter tend to vary too greatly between individuals.

### Implications

The findings of this experimental series are particularly relevant for researchers aiming to measure cognitive load in the context of problem solving. When a task environment, such as a sequence of problem-solving tasks like weekday problems, varies in element interactivity, cognitive load should ideally be assessed at meaningful intervals (i.e., after each task) to capture the dynamic nature of the construct more precisely (Schmeck et al., 2015; Van Gog et al., 2012).

At the same time, researchers should be aware of potential confounding influences, such as heuristic biases, when students evaluate the cognitive load of tasks differing in element interactivity. This may be especially pertinent in cases where there is a strong contrast between an initial task (i.e., the anchor) and subsequent tasks, for example, when students first solve a very easy task followed immediately by a highly complex one. In such instances, anchoring effects may distort subsequent cognitive load assessments.

However, researchers should also be cautious about the frequency of cognitive load assessments, as overly frequent measurement may interfere with students’ engagement in the problem-solving process itself. The extent to which heuristic biases affect cognitive load judgments in more authentic learning contexts remains an open question for future research.

### Limitations and future directions

Apart from the valuable insights provided by this experimental series, there are some limitations that should be considered when interpreting the results and addressed in future research. This experimental series focused primarily on the anchoring effect in students’ assessments of task complexity (i.e., ICL). However, since complexity is only one aspect of CLT, future studies should investigate whether these findings can be replicated when students assess the presentation format (i.e., extraneous cognitive load, ECL) of a task environment. Given that reducing ECL, i.e., cognitive resources that are not directly relevant to actual problem-solving, is a central goal of instructional design (Mayer & Moreno, 2003), future research should investigate whether students are influenced by anchoring when assessing ECL multiple times (e.g., when instructional material varies in ECL at different stages).

Further limitations relate to the problem-solving tasks themselves and the circumstances under which they were examined. Thus, the problem-solving tasks were manipulated in terms of the number of interacting elements, thereby enhancing element interactivity across the three task units. However, the differences in complexity between the three tasks are not equidistant, which should be considered when interpreting the results. Additionally, individual differences in participants’ perceptions of complexity may have influenced their assessments (Brünken et al., 2010). This subjective factor could potentially mask or amplify anchoring effects. Moreover, it remains unclear whether prior familiarity with certain types of tasks affected participants’ complexity ratings, potentially impacting the observed results. Furthermore, the weekday problem-solving tasks may not represent realistic challenges that students typically encounter in their daily university experiences. Therefore, replicating this experimental series in more realistic learning environments (e.g., a classroom setting instead of an online survey) would be valuable for future studies. One potential limitation is that the first problem-solving task was assessed without an internal reference point. Consequently, this initial assessment may be affected by external or individual factors, such as prior knowledge, general experience of the task, or expectations. This introduces interindividual variability.

Concerning the assessment, we acknowledge that extreme ratings of the first task could theoretically restrict the available range for subsequent judgments (i.e., floor or ceiling effects), particularly when the first task is already rated near the lower or upper end of the scale. For example, if the first task is rated as relatively easy (e.g., “2”), but the second task is perceived as even easier, the scale may not allow for a meaningful differentiation. To mitigate this issue, we used a sufficiently wide 9-point rating scale to allow for fine-grained judgments. However, a potential influence of range restriction cannot be entirely ruled out.

In this experimental series, cognitive load was measured using Krieglstein et al.’s (2023) multiple-item scale. In light of the ongoing debate about single-item versus multiple-item scales (Allen et al., 2022) and the argument that multiple assessments of cognitive load during problem-solving may be cognitively fatiguing, future studies could examine whether similar results can be obtained when using a single-item scale, such as the one by Paas (1992). Additionally, incorporating a baseline measure of working memory capacity prior to problem solving and cognitive load assessments could provide valuable insights into whether the assessment of ICL depends on students'ability to hold and process information at any given time.

Moreover, the questionnaire by Krieglstein et al. (2023) may not have been ideally suited for measuring ICL in the context of the three problem-solving tasks, as it was originally developed for use in learning scenarios. For instance, one item was removed from the ICL scale to ensure that prior knowledge was not assessed, focusing instead solely on element interactivity as a defining characteristic of task complexity. Despite this adjustment, internal consistency remained high. Importantly, the observed differences in ICL across the three task units in all experiments provide empirical support for the scale’s sensitivity and thus its validity in detecting variation in perceived task complexity. Nonetheless, future studies should consider employing a questionnaire specifically designed for assessing task complexity in problem-solving contexts. For example, the mental load scale developed by Krell (2017) could serve as a promising starting point for exploring more context-appropriate assessment tools.

Another challenge in assessing cognitive load is the absence of an objective comparison measure to verify the accuracy of assessments. Unlike Tversky and Kahneman’s (1974) studies, where an objective comparison was possible through the provision of a measurable anchor, no such objective measure was available in the experimental series presented here. While the complexity of a task influences cognitive load, it is inherently subjective and lacks a clear, objective benchmark. As a result, the anchor in this study was not an objective reference but rather a subjective assessment of complexity, complicating the comparison of findings with other studies on anchoring effects.

Another limitation concerns the “objective difficulty” of the problem-solving tasks used in this experimental series, as reflected in participants'task performance. It is encouraging that the intended differences in element interactivity were, to some extent, mirrored in the proportion of correctly solved tasks. However, this outcome also highlights certain limitations. While the easiest task unit was solved correctly by nearly all participants, indicating a ceiling effect, the task units with moderate and high element interactivity were sometimes solved at comparable rates (particularly in Experiment 2). In Experiments 1 and 3, task performance was higher for the unit involving moderate element interactivity compared to the unit with higher element interactivity. The relatively large differences in perceived ICL across task units were not always consistently reflected in objective task performance outcomes, suggesting a potential mismatch between subjective and objective measures of task complexity (Makransky et al., 2019; Stark et al., 1998). Another reason for the lack of correlation between ICL assessments and task performance could be that the higher complexity is compensated for by longer processing times resulting from the task’s higher cognitive demands. Future research should therefore aim to manipulate element interactivity with greater precision to ensure that differences in complexity are more reliably reflected in task performance, while simultaneously avoiding floor and ceiling effects.

As previously mentioned, subjective anchors (such as the perception of complexity) appear to have a limited effect on decision-making processes. Future studies could explore whether providing students with prompts indicating that the following task will be easy or difficult (e.g., based on evaluations from other students who have already solved the task) influences both their complexity assessments and task performance. Such prompts might even trigger learning-enhancing processes, for instance, by increasing student engagement based on the anchor information.

## Conclusion

From a measurement perspective, the results of this experimental series suggest that cognitive load assessments do not operate in isolation, especially when administered repeatedly. Rather, they are embedded in a temporal and comparative context. This has important implications for how researchers interpret cognitive load assessments across tasks: tasks of comparable objective complexity may receive different ratings depending on their position within a sequence and the students’ prior impressions. These differences may not reflect actual variations in cognitive load, but rather the students’ tendency to maintain internal consistency or to differentiate between tasks. This interpretation lends weight to the notion that cognitive load assessments are inherently relative (Brünken et al., 2010).

## Data Availability

All data associated with the experiments have been made publicly available on OSF: 10.17605/OSF.IO/G6N5X.

## Appendix A

### Problem-solving tasks

The three problem-solving tasks were adapted from Schmeck et al. (2015).

Lower element interactivity:

Suppose today is Tuesday. What day of the week is the day after tomorrow?

Moderate element interactivity:

Suppose yesterday was Wednesday. What day of the week was 4 days before the day before yesterday?

Higher element interactivity:

Suppose last Tuesday was the second day. What day of the week is it in 17 days, if the eighth day is in 2 days?

## Appendix B

### Intrinsic cognitive load items

The ICL items were taken from the Krieglstein et al. (2023) questionnaire.

The learning content was difficult to understand.

The explanations of the learning content were difficult to understand.

The learning contents were complex.

The learning content included much complex information.

Without prior knowledge, the information was not understandable.*

* The fifth item was not included in the ICL scale.

## Footnotes

1 Complexity is perceived internally and results from the element interactivity, which can be manipulated externally by adding or removing elements in the task (as in the linear equation tasks above).

2 Please note that there are many other situations in which heuristics can be used.

3 Each task unit contained a problem-solving task.

## Declarations

### Open practices statement

None of the experiments reported in this article were preregistered.
